# The function of two P450s, CYP9M10 and CYP6AA7, in the permethrin resistance of *Culex quinquefasciatus*

**DOI:** 10.1038/s41598-017-00486-0

**Published:** 2017-04-03

**Authors:** Youhui Gong, Ting Li, Yucheng Feng, Nannan Liu

**Affiliations:** 1 Department of Entomology and Plant Pathology, Auburn University, Auburn, AL 36849 USA; 2 Department of Crop, Soil and Environmental Sciences, Auburn University, Auburn, AL 36849 USA; 3 Department of Honeybee Protection and Biosafety, Institute of Apicultural Research, Chinese Academy of Agricultural Sciences, No. 1 Beigou Xiangshan, Haidian District, Beijing 100093 P.R. China

## Abstract

Cytochrome P450 monooxygenases play a critical role in insecticide resistance by allowing resistant insects to metabolize insecticides. Previous studies revealed that two P450 genes, *CYP9M10* and *CYP6AA7*, are not only up-regulated but also induced in resistant *Culex* mosquitoes. In this study, CYP9M10 and CYP6AA7 were separately co-expressed with cytochrome P450 reductase (CPR) in insect *Spodoptera frugiperda* (Sf9) cells using a baculovirus-mediated expression system and the enzymatic activity and metabolic ability of CYP9M10/CPR and CYP6AA7/CPR to permethrin and its metabolites, including 3-phenoxybenzoic alcohol (PBOH) and 3-phenoxybenzaldehyde (PBCHO), characterized. PBOH and PBCHO, both of which are toxic to *Culex* mosquito larvae, can be further metabolized by CYP9M10/CPR and CYP6AA7/CPR, with the ultimate metabolite identified here as PBCOOH, which is considerably less toxic to mosquito larvae. A cell-based MTT (3-[4,5-dimethylthiazol-2-yl]-2,5-diphenyltetrazolium bromide) cytotoxicity assay revealed that Sf9 cells expressing CYP9M10/CPR or CYP6AA7/CPR increased the cell line’s tolerance to permethrin, PBOH, and PBCHO. This study confirms the important role played by CYP9M10 and CYP6AA7 in the detoxification of permethrin and its metabolites PBOH and PBCHO.

## Introduction

The mosquito *Culex quinquefasciatus* Say is a primary vector of lymphatic filariasis, St. Louis encephalitis and West Nile encephalitis^1^ and its control is the main strategy for preventing the spread of mosquito-borne diseases, due to a lack of effective vaccines^2, 3^. At present, pyrethroids are by far the most important class of insecticides for the control of mosquito vectors, being used for both insecticide treated bednets (ITNs) and indoor residual spraying (IRS)^4^. However, the rising level of insecticide resistance in *Culex* mosquitoes is rapidly becoming a major problem for mosquito control efforts^5–8^.

Cytochrome P450s are critical for the detoxification and/or activation of xenobiotics and endogenous compounds^9^, through the oxidation in the presence of their obligatory electron donor NADPH cytochrome P450-reductase (CPR), or cytochrome b5 (Cyt b5) on occasion^10^. Transcriptional up-regulation of P450 genes, resulting in increased P450 protein levels and P450 activities, can enhance the metabolic detoxification of insecticides in insects, leading to the development of insecticide resistance. With the development of genome-scale technologies such as oligonucleotide microarrays, next generation sequencing (NGS), suppression subtractive hybridization (SHH), and RNA-seq, multiple over-expressed P450 genes have been identified in resistant mosquitoes^11–19^. The functional characterization of those genes known to be overexpressed in resistance, including *in vitro* recombinant protein metabolism assays and *in vivo* gene silencing and gene knockout using RNAi and CRISPR, respectively, bridges the gap between gene expression and gene function^6, 20–24^. While *E. coli*, yeast, and baculovirus expression systems have been used for the co-expression of P450 and CPR protein complexes from insects^12, 15, 19, 25^ to characterize the function of the P450 genes, baculovirus–mediated insect cell expression systems offer significant advantages for high-level recombinant protein production and processing compound eukaryotic proteins^26–29^. The co-expression of P450s and CPR complexes with baculovirus-mediated expression systems in insect cells has now successfully been used to demonstrate several functional P450 genes in the metabolism of insecticides in insects^30–33^.

The P450 genes CYP9M10 and CYP6AA7 are known to be overexpressed in pyrethroid resistant *Cx. quinquefasciatus* mosquitoes^13, 16–18^. These two P450 genes can be further induced by permethrin in the resistant mosquitoes^17^, suggesting that both constitutive expression and induction of these P450 genes are responsible for the development of resistance in *Culex* mosquitoes. Knockdown of CYP6AA7 and CYP9M10 in resistant *Culex* mosquitoes using RNA interference (RNAi) has confirmed their involvement in insecticide resistance^22, 23^; disrupted CYP9M10 in *Cx. quinquefasciatus* using TALENs and CRISPR^24^ has further demonstrated the role of CYP9M10 in conferring permethrin resistance in Culex mosquitoes. The expression of CYP9M10 in *E.coli* has revealed time- and NADPH-dependent permethrin metabolism^25^, although as yet no metabolites have been identified.

In the current study, a baculovirus-mediated expression system was used to co-express CYP9M10 and CYP6AA7 separately with CPR in Sf9 cells in order to determine whether these two P450s are indeed capable of metabolizing permethrin and its metabolites phenoxybenzyl alcohol (PBOH) and 3-phenoxybenzaldehyde (PBCHO) through P450 oxidation^34^. Prior research has established that oxidation of PBOH and PBCHO in mosquitoes could be mediated by P450s in microsomes, with PBCOOH as the ultimate metabolite in the metabolism of permethrin in mosquito larvae^35^. In particular, two *Aedes aegypti* P450s, CYP6Z8 and CYP6Z2, have been shown to metabolize PBOH and 3- PBCHO, common pyrethroid metabolites produced by carboxylesterases, indicating that the secondary metabolism of pyrethroid insecticides by P450s is also likely to be linked to resistance^26^. These studies imply that P450s are an important factor involved in the metabolic pathway of permethrin in insects. The role of P450 in permethrin metabolism pathway (Fig. S1) in resistant *Culex* mosquitoes are thus clearly in need for further investigation.

In the work reported here, the protein expression conditions were optimized largely based on the catalytic activity of microsomal P450/CYP protein against a7-ethoxycoumarin (7-EC) substrate. A permethrin cytotoxicity assay and HPLC analysis were conducted to identify the effect of permethrin on CYP9M10 or CYP6AA7 and CPR co-expressing cells and to investigate the capacity of the co-expressed P450/CPR microsomal proteins to participate in the metabolism of permethrin and its metabolites. In addition, the toxicity of PBOH, PBCHO and PBCOOH was compared in susceptible and resistant mosquito strains to evaluate the possibility that PBOH and PBCHO are involved in the permethrin resistance. Our results provide direct evidence that CYP9M10/CPR and CYP6AA7/CPR are indeed involved in pyrethroid resistance by metabolizing permethrin insecticides and its metabolites PBOH and PBCHO.

## Results

### P450 content, CPR activity, and P450 activity

**Figure 1:**
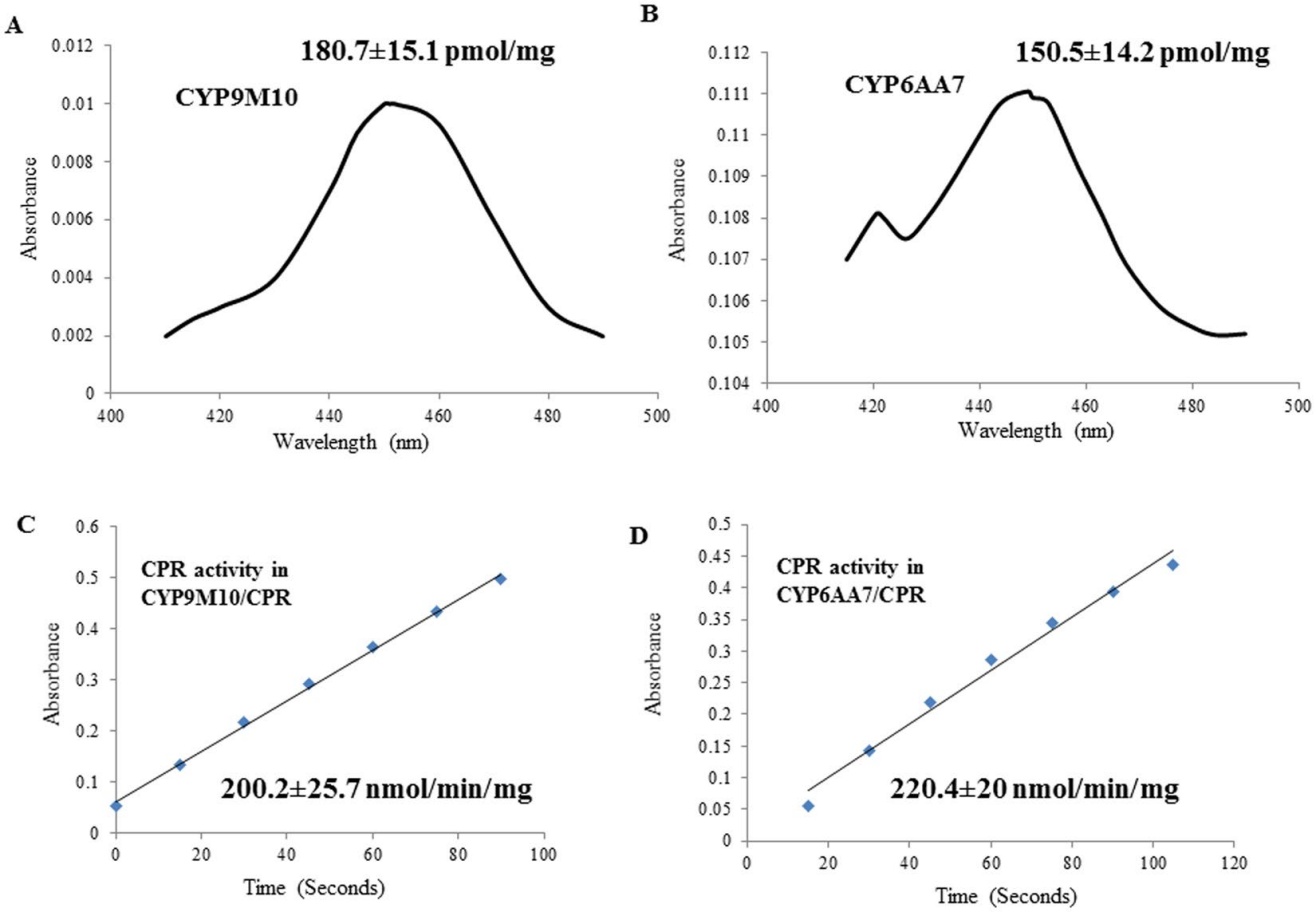
Biochemical characterization of P450 content and CPR activity of co-expressed P450/CPR. (**A**) P450 content detected in microsomal protein of Sf9 cells expressing CYP9M10; (**B**) P450 content detected in microsomal protein of Sf9 cells expressing CYP6AA7; (**C**) CPR activity measured in microsomal protein of Sf9 cells co-expressing CYP9M10/CPR; (**D**) CPR activity measured in microsomal protein of 6AA7/CPR expressing Sf9 cells.

Each of the P450s was co-expressed with CPR by simultaneous infection of Sf9 cells through two recombinant viruses, P450-recombinant baculovirus (P450rbv) and CPR-recombinant baculovirus (CPRrbv). Co-expression of P450 and CPR, the P450/CPR-recombinant baculovirus (P450rbv/CRPrbv), was successfully performed in Sf9 cells with MOI ratios of 0.5 for P450 and 0.05 for CPR in the suspension culture. Sf9 cell co-infection by the P450/CPR-recombinant baculovirus was observed at 72 h post infection (Fig. S2). Microsomal proteins from the co-expressed P450/CPR in Sf9 cells were then isolated and used for the biochemical characterization study. Reduced CO-P450 and CO-P450/CPR both exhibited a characteristic maximum absorption peak at 450 nm, with a P450 content of 180.7 ± 15.1 pmol/mg protein for CYP9M10 and 150.5 ± 14.2 pmol/mg protein for CYP6AA7, showing that CYP9M10 and CYP6AA7 were successfully expressed in the Sf9 cells (Fig. 1A,B). Cytochrome c activity measurements revealing protein activities of 200.2 ± 25.7 nmol/min/mg protein for the co-expressed CYP9M10/CPR and 220.4 ± 20 nmol/min/mg protein for co-expressed CYP6AA7/CPR further confirmed the successful co-expression of CPR in the cells (Fig. 1C,D).

Both the P450/CPR microsomal proteins from the Sf9 cells were found to be capable of converting *in vitro* the substrate 7-ethoxycoumarin to the fluorescent product 7-hydroxycoumarin, with turnover rates (*K*cat) of 21.1 ± 2.2 pmol/min/pmol P450 for CYP9M10/CPR and 26.0 ± 3.4 pmol/min/pmol P450 for CYP6AA7/CPR (Table 1). No significant P450 activity was observed in either the proteins isolated from the original Sf9 cells or those from the cells expressing CYP9M10-, CYP6AA7- or CPR protein alone (Table 1). The V_max_ (maximum velocity) observed in the ECOD activity test were relatively high, at 3818.3 ± 20.3 pmol/min/mg for CYP9M10/CPR and 3895.7 ± 13.5 pmol/min/mg for CYP6AA7/CPR, both considerably above the values measured inSf9 cells of 100.2 ± 34.9pmol/min/mg, CYP9M10 (156.7 ± 31.89 pmol/min/mg), CYP6AA7 (180.5 ± 30.19 pmol/min/mg), and CPR (49.6 ± 14.39 pmol/min/mg), suggesting the importance of P450/CPR co-expression in 7-ethoxycoumarin metabolism (Table 1). The *K*_M_ (Michaelis constant) values for CYP9M10/CPR and CYP6AA7/CPR were found to be 779.3 ± 16.0 µM and 564.76 ± 14.7 µM, respectively. Apparent kinetic parameters like *K*m and *K*cat could not be determined in the Sf9 cells for CYP9M10, CYP6AA7 and CPR alone due to the low P450 activity and content in these microsomes, resulting in the non-michaelian behavior of this substrate for these microsomes (Table 1). The differential efficiency of the substrate metabolism for individual P450s may be due to differences in their structures leading to different substrate specificities.

**Table 1:** ECOD activity of P450/CPR microsomal proteins.

Enzyme source (microsomes)	ECOD activity		
	Vmax (pmol/min/mg)	*Kcat (pmol/min/pmol P450)	K_M_ (µM)
CYP9M10/CPR	3818.3 ± 20.3	21.1 ± 2.2	779.3 ± 16.0
CYP6AA7/CPR	3895.7 ± 13.5	26.0 ± 3.4	564.8 ± 14.7
Parental sf9 cells	100.2 ± 34.9	ND^a^	ND
CYP9M10	156.7 ± 31.8	ND	ND
CYP6AA7	180.5 ± 30.1	ND	ND
CPR	49.6 ± 14.3	ND	ND

*Kcat is the turnover number, calculated as Vmax/[E concentration]. ND^a^: Not Detected.

### Cytotoxicity of insecticides in CYP9M10/CPR or CYP6AA7/CPR-expressing cells (MTT assays)

**Figure 2:**
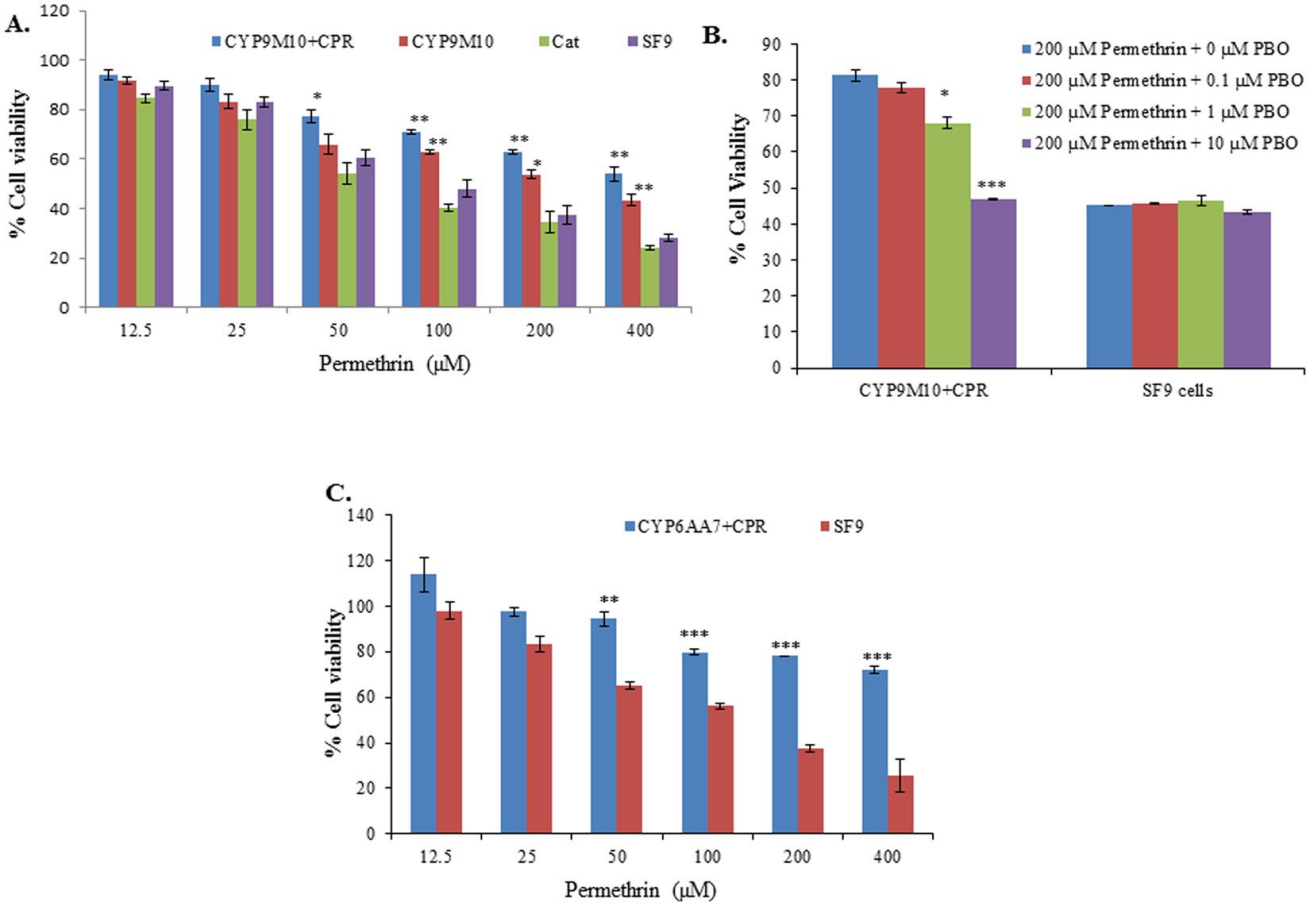
The role of P450/CPR in the detoxification of permethrin in Sf9 insect cells. (**A**) Viability of Sf9 cells co-expressing CYP9M10/CPR, the cells expressing chloramphenicol acetyltransferase (CAT) protein [Invitrogen], and parental Sf9 cells treated with 12.5, 25, 50, 100, 200, and 400 µM of permethrin; (**B**) Effects of permethrin and PBO on viability of co-expressing CYP9M10/CPR Sf9 cells and Sf9 cells alone, treated with 0.1, 1, and 10 µM of PBO; (**C**) Viability of CYP6AA7/CPR co-expressing Sf9 cells and Sf9 cells alone, treated with 12.5, 25, 50, 100, 200, and 400 µM of permethrin. Student’s t-test was used for the statistical significance analysis. *P < 0.05; **P < 0.01; ***P < 0.001.

The cytotoxicity of insecticides was examined for Sf9 insect cells alone and for those containing co-expressed P450-and CPR-recombinant baculovirus. The percentage cell viability of each insecticide concentration was calculated by comparison with that of 0.1% acetonitrile-treated cells. The results revealed that CYP9M10/CPR co-expressing cells were significantly more tolerant to permethrin at concentrations of 100, 200 and 400 µM compared to the two controls, the positive control pENTR™/CAT (plasmid producing baculovirus expressing chloramphenicol acetyltransferase (CAT) protein [Invitrogen]) infected cells and parental sf9 cells (Fig. 2A). As there was no significant difference in the tolerance to permethrin at any of the concentrations tested between the two control cells, in the subsequent MTT experiments we used only parental sf9 cells as the control. We further measured permethrin cytotoxicity in the presence of different concentrations of PBO, a P450 inhibitor, showing that the percentage cell survival against permethrin cytotoxicity in CYP9M10/CPR co-expressing cells was significantly lower when the cells were co-treated with 200 µM permethrin and 1 µM or 10 µM PBO (Fig. 2B). Under the same conditions, the Sf9 control cells showed no change in their permethrin cytotoxicity with PBO treatment (Fig. 2B). This not only indicates that the enhanced cell survival against permethrin cytotoxicity found in cells expressing CYP9M10/CPR was due to the activity of P450s, but also shows that cytotoxicity assays can be a good indicator *in vitro* for determining the ability of cells co-expressing P450/CPR to detoxify insecticides. Similarly, the significant cell viability against the cytotoxic effects of permethrin in CYP6AA7/CPR co-expressing cells demonstrates their enhanced tolerance to permethrin at concentrations of 50, 100, 200 and 400 µM compared to the control cells (Fig. 2C). Taken together, these results suggest a significant role for CYP9M10/CPR and CYP6AA7/CPR in the detoxification of permethrin in insect cells.

**Figure 3:**
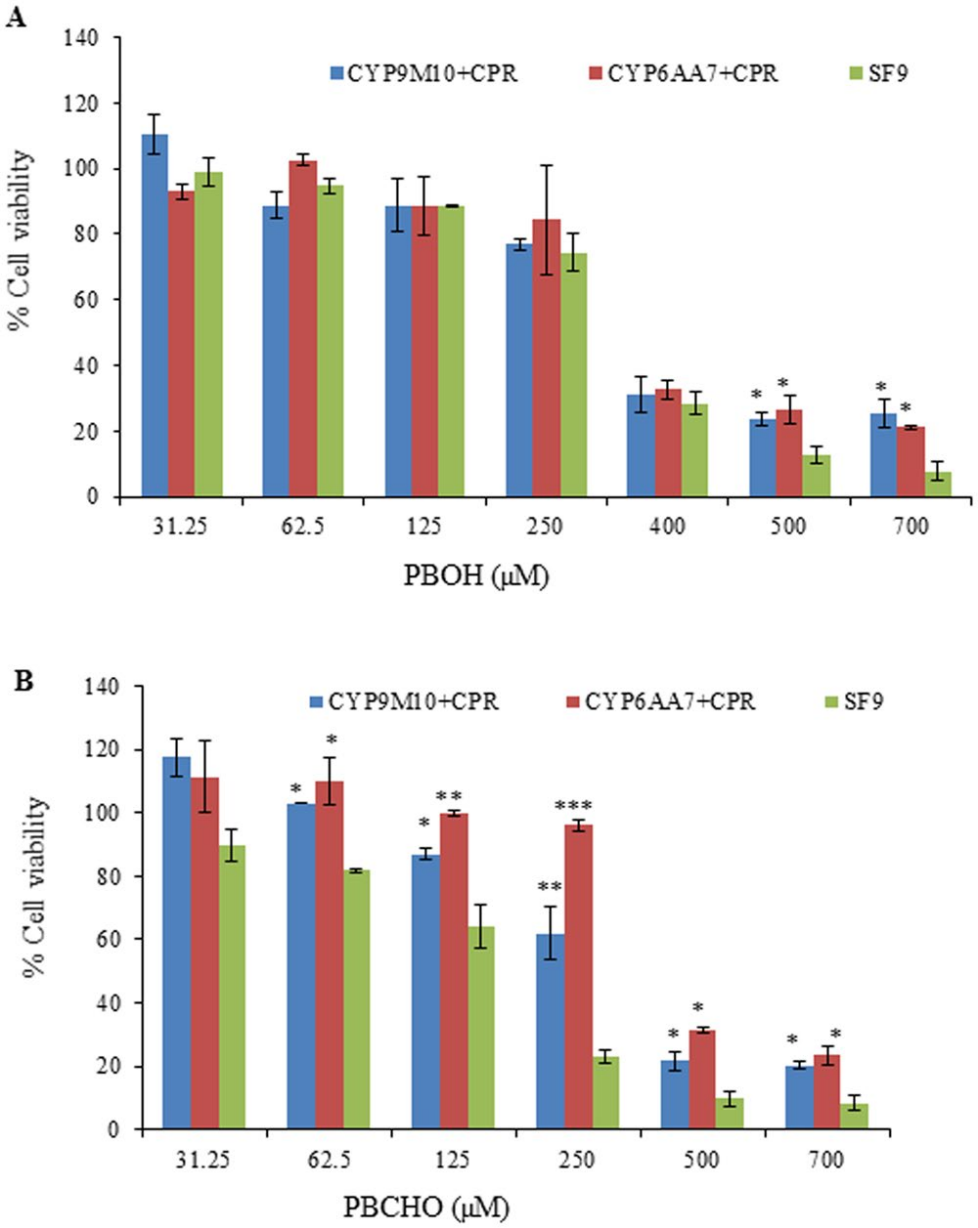
The role of CYP9M10/CPR and CYP6AA7/CPR in the detoxification of PBOH or PBCHO in Sf9 insect cells. (**A**) Viability of CYP9M10 or CYP6AA7 and CPR co-expressing Sf9 cells and Sf9 cells alone, treated with 31.25, 62.5, 125, 250, 400, 500, and 700 µM of PBOH; (**B**) Viability of CYP9M10 or CYP6AA7 and CPR co-expressing Sf9 cells and Sf9 cells alone, treated with 31.25, 62.5, 125, 250, 400, 500, and 700 µM of PBCHO. Student’s t-test was used for the statistical significance analysis. *P < 0.05; **P < 0.01; ***P < 0.001.

The results also show that the co-expression of P450/CPR in Sf9 cells detoxifies both PBOH and PBCHO, the metabolites of permethrin. Sf9 cells are very sensitive to PBOH and PBCHO at concentrations above 400 µM (Fig. 3A). Not only were there significant improvements in cell viability against the cytotoxic effects of PBOH in CYP9M10/CPRand CYP6AA7/CPR co-expression cells observed at concentrations of 500 µM and 700 µM compared to Sf9 cells alone (Fig. 3A), but significantly higher cell viability against the cytotoxic effects of PBCHO in CYP9M10/CPR or CYP6AA7/CPR co-expression cells were also observed at concentrations of 62.5 µM to 700 µM compared to the control cells (Fig. 3B). These results indicate that CYP6AA7/CPR and CYP9M10/CPR may also play an important role in the metabolism of PBOH and PBCHO in insect cells.

### *In vitro* metabolism of permethrin

**Figure 4:**
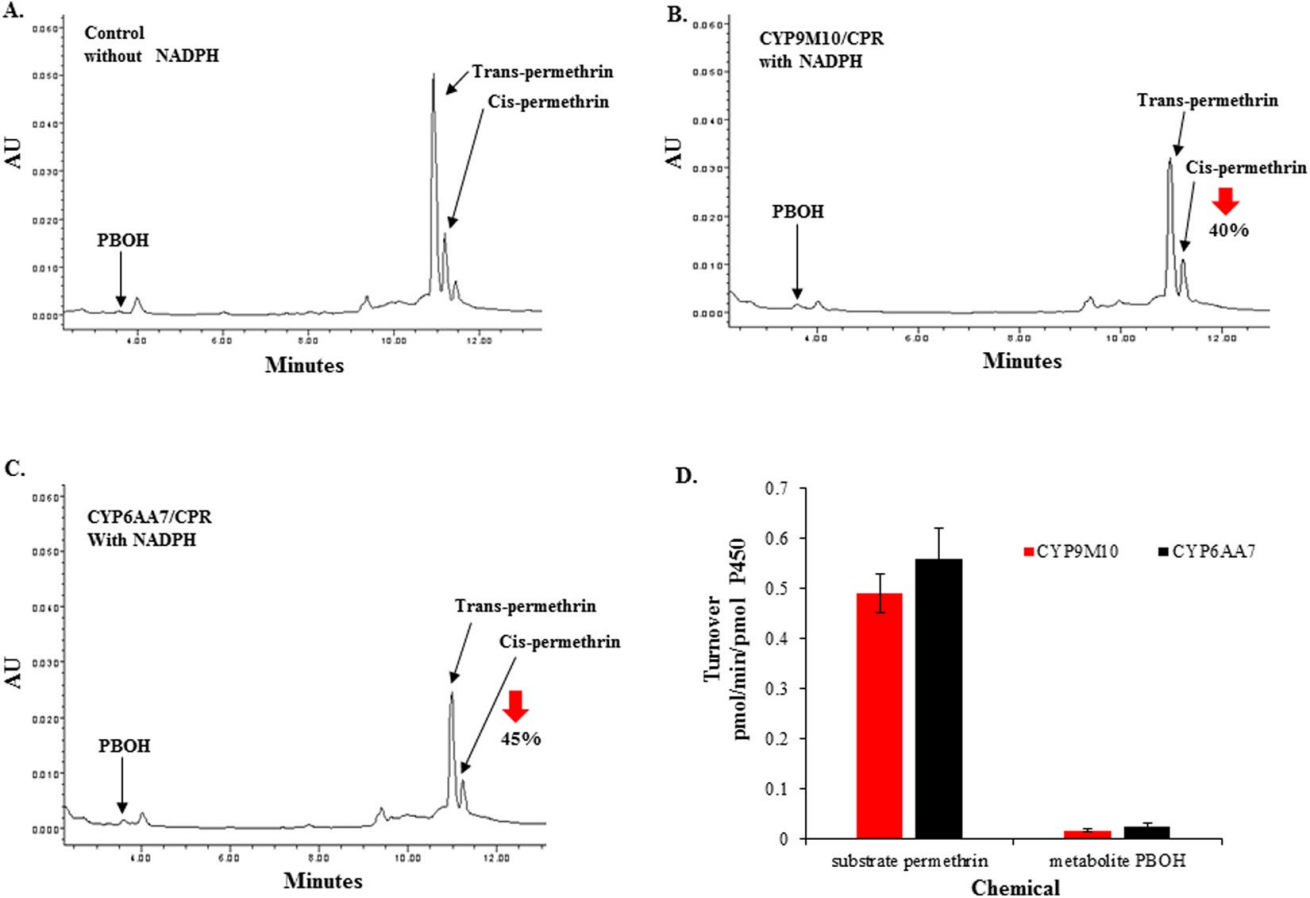
Permethrin metabolism by microsomes of CYP/CPR. (**A**) Control: permethrin (20 µM) incubated with CYP9M10/CPR or CYP6AA7/CPR microsomes (100 pmol P450 in 0.1 M Tris pH = 7.5) for 2 h at 30 °C without NADPH. The black arrows indicate the peaks for PBOH, trans-permethrin, and cis-permethrin; (**B**) Permethrin metabolized by microsomes of CYP9M10/CPR expressing Sf9 cells with NADPH; (**C**) Permethrin metabolized by microsomes of CYP6AA7/CPR expressing Sf9 cells with NADPH; (**D**) Permethrin turnover rate in the CYP/CPR co-expressed microsomal protein with and without NADPH, and the rate of formation of the metabolite PBOH in microsomes of CYP9M10/CPR or CYP6AA7/CPR expressing Sf9 cells.

Permethrin metabolism was assayed with microsomal proteins of CYP9M10/CPR and CYP6AA7/CPR in the presence or absence of NADPH. The degradation of the substrate and the appearance of metabolites were monitored by reverse-phase HPLC. According to the HPLC analysis, trans-/cis-permethrin elution times were 10.7 min and 11 min, respectively (Fig. S3A). Three permethrin metabolites, PBOH, PBCHO and PBCOOH, were used here, with elution times of 3.4, 5.7, and 3.7 min, respectively (Fig. S3B,C,D). The permethrin was significantly metabolized by both CYP9M10/CPR and CYP6AA7/CPR, with 40% and 45% decreases, respectively, in the total 20 µM permethrin compared to the control (without NADPH) after a 120 min incubation period (Fig. 4A,B,C), clearly demonstrating that both CYP9M10/CPR and CYP6AA7/CPR are capable of metabolizing permethrin *in vitro*. CYP9M10/CPR and CYP6AA7/CPR metabolized the permethrin with turnover rates of 0.49 ± 0.04 pmol/min/pmolP450 and 0.56 ± 0.05 pmol/min/pmolP450, respectively (Fig. 4D), similar to the results reported by Müller *et al.* ^12^ and Stevenson *et al*.^15^ for permethrin with *An. gambiae* CYP6P3 and *Ae. aegypti* CYP9J32, CYP9J28, CYP9J26 and CYP9J24, respectively. From the chromatographic analysis, PBOH was eluted at 3.5 min and detected in the presence of NADPH for both CYP9M10/CPR and CYP6AA7/CPR (Fig. 4A,B,C). Based on the chromatographic analysis, the PBOH turnover rate was only 0.016 ± 0.02 pmol/min/pmol P450 for CYP9M10/CPR and 0.025 ± 0.01 pmol/min/pmol P450 for CYP6AA7/CPR (Fig. 4D), indicating that only a small portion of the permethrin was converted into PBOH. These very low turnover rates compared to those for permethrin indicate the likely presence of other metabolites that were not detected under these HPLC conditions.

### *In vitro* metabolism of permethrin metabolites, PBOH and PBCHO

**Figure 5:**
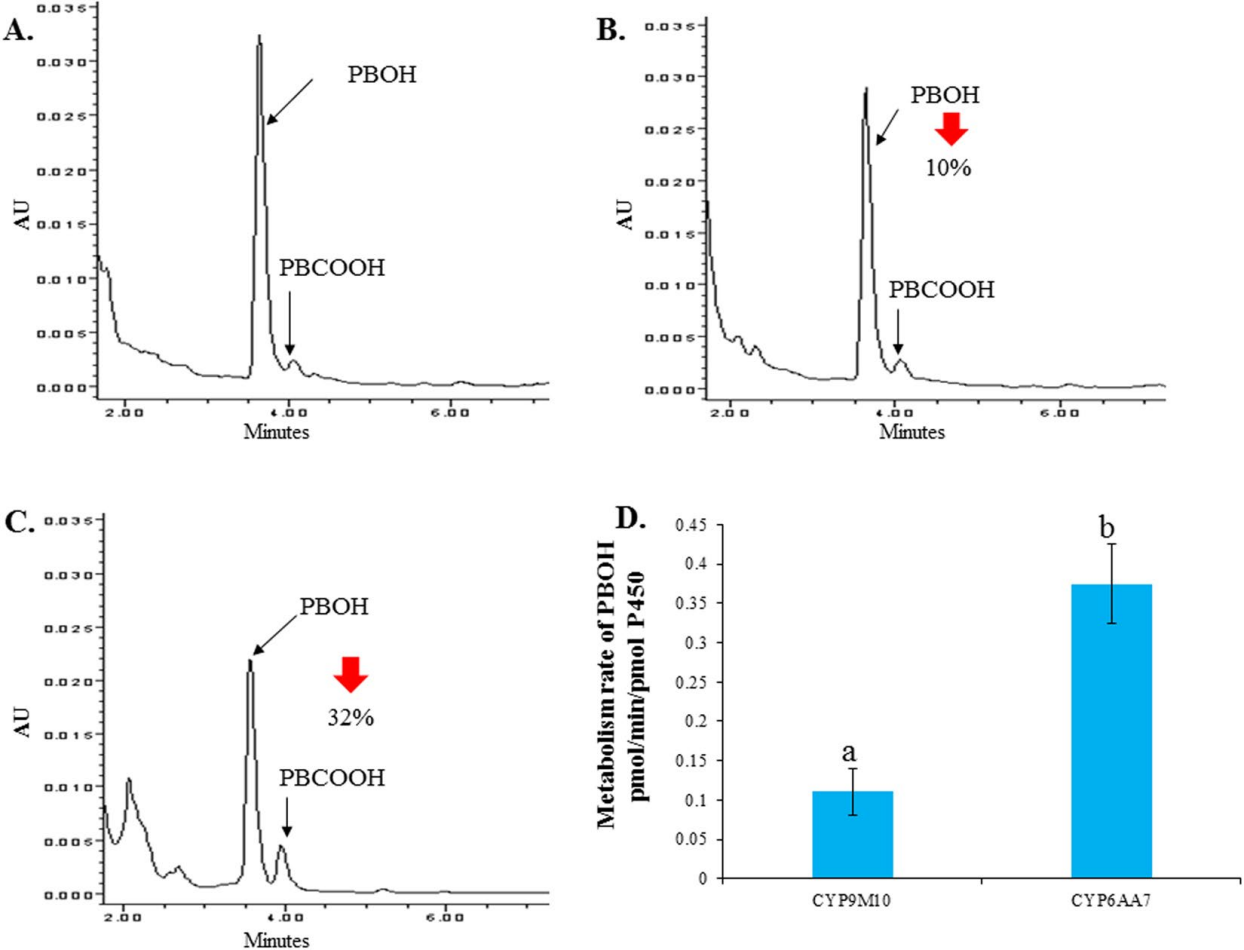
Metabolism profile of PBOH by CYP6AA7/CPR or CYP9M10/CPR microsomes. (**A**) Control: PBOH (20 µM) incubated with CYP9M10/CPR or CYP6AA7/CPR microsomes (100 pmol P450 in 0.1 M Tris pH = 7.5) for 2 h at 30 °C without NADPH. The black arrows indicate the peaks for PBOH and its metabolite PBCOOH; (**B**) PBOH metabolized by microsomes of CYP9M10/CPR expressing Sf9 cells with NADPH; (**C**) PBOH metabolized by microsomes of CYP6AA7/CPR expressing Sf9 cells with NADPH; D. Metabolism rate of PBOH in different CYP/CPR microsomes. The results are shown as the mean ± S.E (n ≥ 3). Statistical significance is represented by P ≤ 0.05 using one-way ANOVA.

When PBOH was used as a substrate (Fig.5A), 10% and 32% decreases in 20 µM PBOH were detected in the presence of NADPH for reactions with CYP9M10/CPR and CYP6AA7/CPR, respectively, compared to the control (Fig. 5B,C). In the presence of NADPH, PBOH was metabolized by CYP9M10/CPR at a rate of 0.11 ± 0.01 pmol/min/pmolP450 and metabolized by CYP6AA7/CPR at a rate of 0.37 ± 0.01 pmol/min/pmolP450 (Fig. 5D). The presence of the metabolite PBCOOH was detected based on a comparison with the chromatographic profile obtained for a PBCOOH standard (Fig. 5A,B,C).

**Figure 6:**
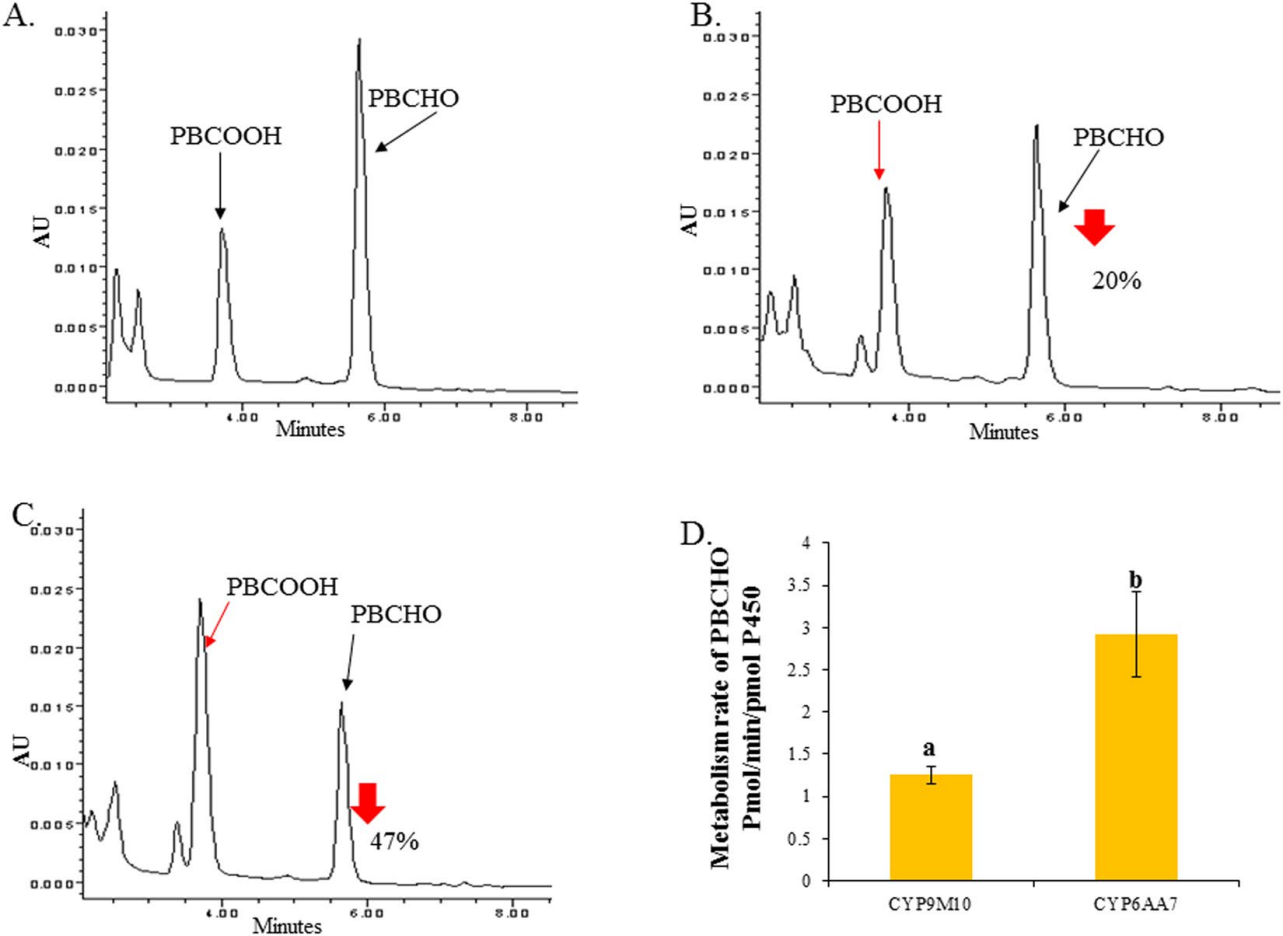
Metabolism profile of PBCHO by CYP6AA7/CPR and CYP9M10/CPR microsomes. (**A**) Control: PBCHO (10 µM) treated with CYP/CPR microsomes (100 pmol P450 in 0.1 M Tris pH = 7.5) for 15 min at 30 °C without NADPH. The black arrows indicate the peaks for PBCHO and PBCOOH; (**B**) PBCHO metabolized by CYP9M10/CPR microsomes. The red arrows indicate the PBCOOH and the reduced amount of PBCHO; (**C**) PBCHO metabolized by CYP6AA7/CPR microsomes. The red arrows indicate the PBCOOH and the reduced amount of PBCHO; (**D**) Metabolism rate of PBCHO with different CYP/CPR microsomes. Statistical significance is represented by P ≤ 0.05, with the letters using one-way ANOVA.

When PBCHO was used as a substrate (Fig. 6A), PBCHO was degraded by about 20% by CYP9M10/CPR and 47% by CYP6AA7/CPR in the presence of NADPH over the course of a 15 min reaction, compared to the control (Fig. 6B,C). The metabolic rates were 1.3 pmol/min/pmolP450 for CYP9M10/CPR and 2.7 pmol/min/pmol P450 for CYP6AA7/CPR (Fig. 6D). PBCOOH was again identified as the major metabolite.

### Toxicity of permethrin metabolites in *Culex* mosquito larvae

*In vitro* metabolism assays with microsomes extracted from *Ae. aegypti* larvae have suggested that PBOH and PBCHO can be transformed to PBCOOH, the ultimate metabolite in the metabolism of permethrin in mosquito larvae^35^. However, the relative toxicity of each metabolite is not yet clear. In order to determine the toxicity of PBOH, PBCHO, PBCOOH and their specific roles in the development of permethrin resistance in *Culex* mosquitoes, we conducted bioassays to compare the toxicities of permethrin, PBOH, PBCHO and PBCOOH in susceptible and resistant mosquito strains. Permethrin toxicity was found to be similar to that identified in a previous study by our group^36^, while PBOH and PBCHO had higher toxicities in mosquito larvae compared to PBCOOH. The permethrin resistant *Culex* strain HAMCq^G8^ showed ~3-fold greater resistance to PBOH and PBCHO compared to the permethrin susceptible strain S-Lab, but there was no difference in the toxicity of PBCOOH for the HAmCq^G8^ and S-Lab strains (Table 2).

**Table 2:** Toxicity of Permethrin, PBOH, PBCHO and PBCOOH to the larvae of *Culex quinquefasciatus*.

Insecticide	Strain	LC_50_ (ppm)	95% CI^a^	RR^b^
Permethrin	S-Lab	0.007	0.005–0.08	1
	HAmCq^G8^	19.00	10–33	2714.3
PBOH	S-Lab	16.21	14.06–18.69	1
	HAmCq^G8^	55.03	42.10–71.93	3.4
PBCHO	S-Lab	14.37	11.15–18.52	1
	HAmCq^G8^	48.64	40.00–59.10	3.4
PBCOOH	S-Lab	296.36	268.78–326.77	1
	HAmCq^G8^	289.82	263.39–318.90	0.98

^a^95% confidence interval; toxicity of insecticide is considered significantly different when the 95% CI fail to overlap. ^b^RR: LC_50_ of the resistant strain/LC_50_ of the S-Lab strain.

## Discussion

CYP9M10 and CYP6AA7 have been linked to permethrin resistance in *Cx.quinquefasciatus* in a number of previous studies^13, 18, 22, 23^. Although the overexpression of P450 genes has been widely identified in insecticide resistant insects, studies of catalytic activity against insecticides are generally limited. *In vitro* expression of functional P450 proteins is very important for reconstituting enzymatic activity against substrates and investigations of the role of P450 proteins in the metabolism of insecticides or other xenobiotics. Although *E. coli* and yeast-mediated P450 expression systems are commonly used to express P450s and have higher yields, some eukaryotic P450s are intractably expressed in these expression systems^26^. Baculovirus-mediated expression systems therefore serve a useful purpose by producing P450s with high levels of activity, native CYP enzymes that combine eukaryotic cellular and redox machinery with an organelle structure that is appropriate for the translation of eukaryotic P450s; they have been successfully used to express and characterize various P450s found in insects^30–33, 37^. This study presents the successful co-expression of mosquito P450s with co-factor CPR in Sf9 cells using a baculovirus-mediated expression system, with co-expressed CYP9M10/CPR and CYP6AA7/CPR both exhibiting excellent ECOD activity. This expression profile could thus offer a new expression mode for other P450s, although it is possible that the cytochrome P450 reductase might have different affinities with different P450 proteins. To our knowledge, this is the first time co-expressed *Cx*. *quinquefasciatus* P450 protein and its co-factor CPR have been developed in a baculovirus-mediated expression system.

In this baculovirus expression system, each P450 gene was co-expressed with CPR by the simultaneous infection of sf9 cells by two recombinant viruses, P450rbv and CPRrbv. This co-infection strategy has been explored in studies of human CYP1A1^29^ as well as insect P450s such as CYP9T2 in *Ips pini* ^37^, CYP6BQ9 in *Tribolium castaneum* ^30^, CYP6CM1 in *Bemisia tabaci* ^32^ and CYP6BQ23 in *Meligethes aeneus* ^33^. In baculovirus-mediated expression systems, suitable MOI ratios for the gene-recombinant baculovirus co-infections are not only determined by the protein production and toxicity of the virus in the cells^38^, but also by the enzymatic catalytic activity, which depends on properly posttranslational modified proteins accumulating in the right ratio as a result of primary, secondary and, possibly, tertiary infections of both viruses^39, 40^. The co-expression of P450 and CPR in Sf9 cells has been studied with different MOI ratios of P450 to CPR in baculovirus expression systems^31–33, 41, 42^. Here, we took advantage of the ECOD activity assay to determine the optimum MOI ratio of *CYP9M10-*and *CPR-*recombinant baculovirus for our experiment, which was 0.5:0.05. Similar ratios were recommended by Chen *et al*.^39^ (0.015:0.0015) and Zhu *et al*.^30^ (1.0:0.1), strongly supporting the results reported here and implying that the ECOD activity truly represents the best method for measuring functional P450 expression. However, different P450s may have preference for different standard substrates. For instance, CYP6BQ23 prefers bulkier molecules such as 7-benzyloxymethoxy-4-trifluoromethyl coumarin (BOMFC), whereas well-known standard substrates such as 7-ethoxy coumarin were not significantly metabolized^33^.

CYP9M10 and CYP6AA7 are overexpressed in *Culex* permethrin resistant mosquitoes^13^ and can also be induced by permethrin exposure in resistant strains^17, 43^. The function of these two P450 genes has already been investigated via RNAi, revealing their important role in conferring permethrin resistance^23^. Furthermore, by using the genome editing technologies TALEN and CRISPR/Cas9, Itokawa *et al*.^24^ disrupted *CYP9M10* gene function in a resistant strain of *C. quinquefasciatus* by reducing the pyrethroid resistance of the disrupted *CYP9M10* haplotype line, providing strong evidence that *CYP9M10* is a major factor responsible for the strong pyrethroid resistance in the JPP resistant strain. In the present study, heterogeneously expressing these two P450 genes in a baculovirus expression system and reconstituting catalytically functional recombinant enzyme activity against permethrin and its metabolites PBOH and PBCHO provided an additional way to validate the functional roles of these two P450 genes in permethrin resistance, directly supporting the contention by Li *et al*.^23^ and Itokawa *et al*.^24^ that CYP9M10 and CYP6AA7 play a role in the permethrin resistance of *Cx. quinquefasciatus*.

Expressing CYP9M10 in an *E. coli* system confirmed the ability of recombinant CYP9M10 to metabolize permethrin^25^, clearly demonstrating both time- and NADPH-dependent permethrin metabolism. However, Wilding *et al.* did not validate the known metabolite/metabolites of permethrin metabolism in *Cx. quinquefasciatus*. In the current study, we observed a decreased level of permethrin following P450 metabolism and similar results have been reported in *An. gambiae* CYP6P3^12^. Although cytochrome P450 is known to play an important role in the oxidative metabolic reactions of PBOH to PBCHO and PBCHO to PBCOOH in rats^34^, PBOH was the only metabolite of permethrin detected in our study using high performance liquid chromatography. Although hydroxylation of aromatic rings or methyl groups of permethrin by cytochrome P450 has been demonstrated in previous studies, de-esterification catalyzed by both esterases and cytochromes P450 is also known to be a route for the detoxification of pyrethroids in insects^44^. Casida and Ruzo^45^ and Funaki *et al*.^46^ have argued that ester cleavage to 3-phenoxybenzyl alcohol (PBOH) generally proceeds via an oxidative mechanism. Interestingly, Boonsuepsakul *et al*.^47^ reported that 3-phenoxybenzaldehyde (PBCHO) was a major product of CYP6AA3-mediated deltamethrin metabolism, measured by combined GC-MS analysis. The difference between our study and the study by Boonsuepsakul *et al*.^47^ may be due to the low detoxification rate of PBOH by CYP9M10 and CYP6AA7, which will limit the further oxidation of PBOH to PBCHO. In this study, PBCHO was found to be further oxidized to PBCOOH both by CYP9M10 and CYP6AA7 in the presence of NADPH in the reactions. However, without NADPH, only very low levels of PBCOOH were detected by HPLC analysis. This result might be due to the self-degradation of PBCHO in aqueous reactions or the oxidation of PBCHO to PBCOOH mediated by the ALDHS (aldehyde dehydrogenase) present in the expressed CYP microsomes. The ability of ALDHS to oxidize PBCHO to PBCOOH has been demonstrated in humans^48^ as well as in *A. aegypti* mosquitoes by Somwang *et al*.^35^. Taken together, our baculovirus expression and metabolism assays strongly suggest the importance of *Culex* CYP9M10 and CYP6AA7 in the permethrin metabolic pathways.

These metabolism assay results are backed up by our cell toxicity assays. The cell based MTT assay results presented here demonstrate that the co-expression of CYP9M10/CPR or CYP6AA7/CPR in Sf9 cells can elevate the cell’s ability to tolerate permethrin treatment and even treatments with its metabolites PBOH and PBCHO, thus indirectly supporting the role of these two P450 genes in the catabolism of permethrin metabolic pathways. Duangkaew *et al*.^49^ also used MTT assays to prove the capability of *Ae.aegypti* CYP6P7 and CYP6AA3 in detoxifying cytotoxic pyrethroids in Sf9 cells.

In our study, PBOH and PBCHO were found to be much more toxic to mosquitoes than PBCOOH, indicating that the metabolism of PBOH and PBCHO to PBCOOH is very important for the survival of mosquitoes. The three fold higher resistance to PBOH and PBCHO in permethrin resistant mosquitoes compared to a susceptible strain indicates that resistant mosquitoes can tolerate higher levels of PBOH and PBCHO due to their ability to metabolize PBOH and PBCHO into PBCOOH, demonstrating the importance of the ability of CYP9M10 and CYP6AA7 to metabolize permethrin and the secondary metabolites of PBOH and PBCHO.

In conclusion, the new CYP9M10 and CYP6AA7 reconstitution system reported here enabled us to investigate the role played by CYP9M10 and CYP6AA7 in metabolizing permethrin and its metabolites *in vitro*. This ability of CYP9M10 and CYP6AA7 to metabolize permethrin and PBOH and PBCHO supports the finding that overexpressed CYP9M10 and CYP6AA7 are likely to be a causative factor in the development of pyrethroid resistance in the *Cx. quinquefasciatus* mosquito.

## Materials and Method

### Mosquito strains

Two mosquito strains of *Cx. quinquefasciatus* were used in this study. HAmCq^G8^ is the eighth generation of permethrin-selected offspring of the HAmCq^G0^ strain, which was collected from Madison County and Mobile County, AL^36^; S-Lab is an insecticide susceptible strain kindly provided by Dr. Laura Harrington (Cornell University, Ithaca, NY). HAmCq^G8^ has a 2700-fold higher resistance to permethrin compared with S-lab. All the mosquitoes were reared at 25 ± 2 °C under a photoperiod of 12: 12 (L: D) h (insectary conditions) and fed blood samples from horses (Large Animal Teaching Hospital, College of Veterinary Medicine, Auburn University, Auburn, AL).

### Construction of pENTR™ expression plasmids to obtain C*YP9M10*, *CYP6AA7* and *CPR* genes

Total RNA was extracted from 4^th^ instar larvae of HAmCq^G8^ using the acidic guanidine thiocyanatephenol-chloroform method^50^. The DNA was removed from the total RNA of each mosquito sample using DNase (TURBO DNA-free, Ambion) and the DNA-free total RNA was reverse-transcribed to cDNA using a Transcriptor First Strand cDNA Synthesis kit (Roche) and odigo-dT primer following the manufacturer’s instructions. The gene-specific primers used for cloning the complete reading frames of the *CYP9M10*, *CYP6AA7*and *CPR* genes were designed according to the *Cx. quinquefasciatus* genome sequence^1^ (https://www.vectorbase.org/organisms/culex-quinquefasciatus) (Table 3). Four nucleotide bases *CACC* were added to the 5′ end forward primer (immediately upstream of the ATG transcription start codon. Table 3), enabling the P450 products to be directly cloned into the pENTR™ TOPO® vector (Invitrogen) by annealing to the CACC sequence in the PCR products with the overhang sequence GTGG in the vector. The recombinant vector was then transformed into One Shot® competent *E. coli*. pENTR™ plasmids with *CYP9M10*, *CYP6AA7*, or *CPR* and purified using the PureLink HQ Mini plasmid purification Kit (Invitrogen). Orientation of the inserted genes was tested by PCR using the forward primer of each specific gene and the reverse primer of M13, as per the manufacturer’s instructions (Invitrogen). Expression plasmids were further sequenced for validation.

**Table 3:** Primers used for cloning P450 genes and *CPR*.

Gene	Gene ID*	Orientation	Sequence
*CYP9M10*	CPIJ014218	forward	5′ CACC**ATG**ACCTCACTCGAGTGGTTG 3′
		reverse	5′ TCATTTCTTCATCTCCCCTCTCGG 3′
*CYP6AA7*	CPIJ005959	forward	5′ CACC**ATG**TCTCTGCTGAACACGCT 3′
		reverse	5′ TCACTCTTTGGAGTTGATTTTGTTCAC 3′
*CPR*	CPIJ014664	forward	5′ CACC**ATG**GACGCACAGACAGAGCC 3′
		reverse	5′ ACTCCACACGTCGGCCGAGTACCG 3′

*https://www.vectorbase.org/organisms/culex-quinquefasciatus.

### Recombinant baculovirus expression of CYP9M10, CYP6AA7, and CPR in Sf9 cells

Recombinant baculovirus containing P450 or CPR gene was constructed by incubating pENTR™ expression plasmids of *CYP9M10*, *CYP6AA7*, or *CPR* with BaculoDirect Linear DNA and LR clonase^TM^ II enzyme mix using the BaculoDirect™ Baculovirus Expression system, incubated overnight at 25 °C as per the manufacturer’s instructions (Invitrogen). Recombinant baculovirus containing *CYP9M10*, *CYP6AA7*, or *CPR* was tested by polymerase chain reaction (PCR). The recombinant baculovirus was then transfected into *Spodopterafrugiperda* (Sf9) cells using CellfectinR II Reagent (Invitrogen) to produce recombinant baculovirus stock solutions of SF900-III medium containing 10% FBS and Grace’s insect medium. The preparation of large-scale high titer stocks of recombinant baculovirus for the expression of proteins in insect cells was performed according to the manufacturer’s instructions (Invitrogen). The baculovirus titer was measured by plaque forming assay and a titer of ~2 × 10^8^ pfu/mL used as the final stock for infection of the Sf9 cell in the large scale amplification of expressed CYP9M10, CYP6AA7, or CPR proteins. To co-express P450 and CPR (CYP/CPR) in Sf9 cells, the final stocks of P450- and CPR- recombinant baculovirus were co-infected in Sf9 cells and 1 µg/mL hemin and 0.1 mM 5-ALA, which had been shown to yield the highest catalytic activity of P450 by a coumarin 7-hydroxylase activity assay (ECOD assay, Fig. S4), added to the culture medium 24 h after Sf9 cell co-infection. The optimum multiplicity of infection (MOI) ratio of 10:1 for the co-expression of the CYP/CPR-recombinant baculovirus in Sf9 cells was determined according to the highest P450 activity found in the ECOD assay (Fig. S5).

### Preparation of microsomal proteins

Whole cell lysate protein was collected 72 h after co-infection. The cells were prepared by pelleting them at 1000 × g for 10 min at 4 °C, followed by washing twice with ice-cold PBS buffer (pH 7.4), and re-suspension in homogenization buffer (0.1 M phosphate (pH 7.4), 1.0 mM EDTA, 0.25 M sucrose and 0.5 mM PMSF). They were then homogenized by sonication for 12 seconds, after which the crude homogenate was centrifuged at 8000 × g for 10 min at 4 °C followed by ultracentrifugation (SORVAll Discovery 90SE) at 370,000 × g for 60 min at 4 °C. The resulting microsomal protein pellets were re-suspended in resuspension buffer (0.1 M phosphate buffer pH 7.4, 20% glycerol buffer, 1 mM EDTA, 0.1 mM DTT, 1 mM PMSF) containing a protease inhibitor cocktail and stored at −80 °C until use.

### P450 content and activity determination

To assay the P450 content, microsomal protein was measured and analyzed using a UV/visible spectrophotometer (DU640, Beckman Coulter, USA) according to the procedure described by Liu and Scott^51^. P450-mediated activity was estimated by measuring 7-ethoxycoumarin O-deethylase^52, 53^ with a slight modification. Briefly, a 50 μL enzyme solution (containing 10 μL microsomal fraction and 40 μL of sodium phosphate buffer, 0.1 M, pH 7.5), 40 μL of sodium phosphate buffer (0.1 M, pH 7.5), and 1 μL of 40 mM 7-ethoxycoumarin in acetone were added to each well of a 96-well microplate. The reaction was started by adding 10 μL of aqueous 10 mM NADPH to each well, resulting in final concentrations of 1 mM of NADPH and 0.4 mM of 7-EC. The plate was then incubated for 30 min at 30 °C with shaking. The self-fluorescent NADPH was removed by adding 10 μL of oxidized glutathione (30 mM in water) and 10 μL of glutathione reductase (total 0.5 U). After 10 min incubation at room temperature, the reaction was stopped by adding 120 μL of 50% acetonitrile in TRIZMA-base buffer (0.05 M, pH 10). The 7-hydroxycoumarin formation (excitation: 390 nm, emission: 465 nm) was measured at room temperature with a 96-well microplate reader (Cytation 3 imaging reader, BioTekUSA). A non-enzymatic reaction without microsomes served as the control. A standard curve for 7-hydroxycoumarin was used to calculate the product formation rate. For the determination of the kinetic parameter, the substrate concentrations ranged from 0.1 to 1.2 mM.

### CPR activity determination

CPR activity was determined using the method recommended by Liu and Scott^51^ with slight modifications. A cytochrome c assay was used to determine the activity of CPR. Briefly, microsomal protein was placed in a 1 cm optical path cuvette to which 500 nM cytochrome c was added and the baseline recorded. The initial rate of the cytochrome c reduction was monitored at 550 nm for 3 min after 0.2 mM NADPH was added and immediately mixed, using a UV/visible spectrophotometer (DU640, Beckman Coulter, USA). Activity is expressed here as units per milligram protein, with one unit of reductase activity defined as 1 nmol cytochrome c reduced per minute.

### MTT cytotoxicity assay

The cytotoxicity assay was conducted according to Boonsuepsakul *et al*.^47^ with modifications. Briefly, cells co-expressing P450- (MOI = 0.5) and CPR-recombinant baculovirus (MOI = 0.05) were cultured in 25 cm^2^ flasks. Controls containing no baculovirus-infected cells utilized original Sf9 cells. P450/CPR-recombinant virus expression cells or Sf9 cells alone were seeded onto 24 well plates with a density of 2 × 10^5^ cells/well. For the MTT assays, control cells and infected cells were treated with either permethrin concentrations (diluted in 0.1% acetonitrile) ranging from12.5 to 400 µM, or the permethrin metabolites PBOH and PBCHO (over the range from 12.5 to 700 µM) at 48 h post infection. Permethrin at concentrations ≥500 µM was not used due to its limited solubility^49^. The cytotoxic effects of the insecticides were evaluated by MTT assays using a MTT cell viability assay kit (Sigma). After 48 h insecticide treatment, the medium was removed and the cells washed with PBS buffer (0.1 M, PH 7.4), after which 200 µl MTT (5 mg/ml) was added and the plate incubated at 37 °C for 4 hours. The MTT assay was measured at OD 540 nm on the Cytation 3 imaging reader (BioTek, USA). The experiments were performed with at least three replicates in different wells. Cell survival rates were calculated as (OD value of permethrin treated cells/OD value of acetonitrile treated cells) × 100%. Each treatment was repeated at least 3 times with independent protein expression in the Sf9 cells. For the inhibition assay, the inhibitor piperonyl butoxide (PBO) was added (at 0.1, 1, and 10 µM) with 200 µM permethrin and the results compared with those for permethrin alone.

### HPLC analysis and permethrin, PBOH, and PBCHO metabolism study

Permethrin, PBOH and PBCHO (HPLC grade, Sigma Aldrich) were initially dissolved in acetonitrile and PBCOOH (HPLC grade, Sigma Aldrich) in methanol to create 1 mM standard solutions. Serial dilutions were then prepared of permethrin, PBOH and PBCHO in TrisHCl buffer (pH 7.4)/acetonitrile (1:1, v/v) and PBCOOH in TrisHCl buffer (pH 7.4)/methanol (1:1, v/v) to create a set of standard curves.

The *in vitro* reactions contained 20 µM permethrin and either 20 µM PBOH or 10 µM PBCHO, 100 pmol of either CYP9M10/CPR or CYP6AA7/CPR microsomes, 0.25 mM MgCl_2_, and 1 mM NADPH for a total reaction volume of 700 uL; NADPH was omitted in the control reaction. After 2 h incubation (15 min for PBCHO metabolism) at 30 °C with 60 rpm shaking, the reactions were stopped by adding 700 μl of acetonitrile or methanol to the flasks containing permethrin and either PBOH or PBCHO, incubating for a further 20 min with 100 rpm shaking, and then centrifuged for 10 min at 16,000 × g to pelletize the proteins. The supernatant was collected via filtration and transferred into ultraclean glass vials for reverse-phase HPLC analysis. Reactions were performed in triplicate and a paired T-test of sample reactions (+NADPH) vs the control (−NADPH) performed for the statistical measurements of substrate depletion. Based on the standard curves previously prepared for permethrin, PBOH, and PBCHO, the turnover rates of the substrates and products for the 2 h or 15 min reactions were also calculated and the results expressed as pmol substrate (or product)/min/pmol P450. The individual metabolism rates for permethrin, PBOH, and PBCHO were monitored by reverse-phase HPLC using an HPLC system (Alliance Waters 2695) equipped with a Nova-Pak C18 column (60 Å, 4 µm, 3.9 mm × 150 mm, 1/pkg [WAT086344]) and a Waters 2487 Dual λ absorbance detector.

Two solvents (solvent A: 90% acetonitrile and 10% H_2_O, solvent B: 95% water, 5% acetonitrile adjusted to pH 2.3 with 85% phosphoric acid) were used for the gradient elution (flow rate: 1 ml/min). The gradient system (linear increase) was initially 50% of solvent A and 50% of solvent B rising to 75% of solvent A at 6 min and finishing at 100% of solvent A at 8 min. The flow of 100% solvent A was maintained for a further 4 min and then reduced to 50% at 13 min and maintained for a further 4 min to prepare the column for the next run (method according to Choi *et al*.^48^, with some modifications). The chromatographic analysis was conducted at 23 °C and monitored by absorbance at 232 nm. The insecticide was quantified by peak integration and calculated based on the standard curves prepared previously.

### Toxicity studies of permethrin, PBOH, PBCHO, and PBCOOH - bioassays

To investigate the toxicity of permethrin, PBOH, PBCHO, and PBCOOH against the permethrin resistant (HAmCq^G8^) and susceptible (S-Lab) *Culex* mosquitoes, larval bioassays were conducted. Serial dilutions of permethrin from 0.001–30 ppm and PBOH and PBCHO from 90–500 µM were prepared using tap water with 1% acetonitrile; serial dilutions of PBCOOH from 500 µM–2000 µM were prepared in tap water with 1% methanol. The bioassay method was as described in our previous studies^36, 54^, with minor modifications. Each sample consisted of 10 fourth-instar mosquito larvae in a 10-mL glass tube with 5 mL of the insecticide solution; five concentrations that resulted in 0 to 100% mortality were conducted for each bioassay, each concentration was repeated 3 times. Control groups received only 1% acetonitrile or methanol. Mortality was assessed after 24 h. Each bioassay was repeated three times, each on a different day, and the data were pooled and analyzed using a standard probit analysis, as described by Liu *et al*.^54^ with a computerized version of Raymond^55^. Statistical analysis of LC_50_ values was based on non-overlapping 95% confidence intervals (CI). Resistance ratios (RRs) were calculated as the LC_50_ of the resistant strain divided by the LC_50_ of the susceptible strain.

## supplementary material

The function of two P450s, CYP9M10 and CYP6AA7, in the permethrin resistance of Culex quinquefasciatus
