# Supplementary material for: The function of two P450s, CYP9M10 and CYP6AA7, in the permethrin resistance of Culex quinquefasciatus
Source: Sci Rep. 2017 Apr 3;7:587. doi: 10.1038/s41598-017-00486-0 (PMC5428437; doi:10.1038/s41598-017-00486-0)
Supplement: Supplementary file 1 — The function of two P450s, CYP9M10 and CYP6AA7, in the permethrin resistance of Culex quinquefasciatus [file 41598_2017_486_MOESM1_ESM.pdf]

The function of two P450s, CYP9M10 and CYP6AA7, in the permethrin resistance of *Culex quinquefasciatus*

Youhui Gong[1,2], Ting Li[1], Yucheng Feng[3], Nannan Liu[1]*

**Supplement Information**

**Figure legends**

Figure S1. The hypothetical role of CYPP450 in permethrin metabolism pathway in *Culex quinquefaciatus*. The metabolic pathway of permethrin is proposed in this study according to studies by Nakamura et al.[34], Somwang et al.[35] and Chandor-proust et al.[26].

Figure S2. Sf9 cell co-infection observed at the 72h post infection. A, cells co-infected by empty linear virus; B, cells co-infected by *CYP9M10*- and *CPR*-recombinant baculovirus; C, cells co-infected by *CYP6AA7*- and *CPR*-recombinant baculovirus.

Figure S3. Permethrin and its metabolites in HPLC analysis. A, trans-/cis- permethrin was eluted at 10.7 and 11 min, respectively; B, PBOH was eluted at 3.4 min; C, PBCHO was eluted at 5.7 min; D, PBCOOH was eluted at 3.7 min.

Figure S4. Effects of heme precursors on CYP9M10 ECOD toward 7-ethoxycoumarin. Sf9 cells were infected with *CYP9M10*- and *CPR*-recombinant baculovirus (MOI: 2:1) with 5 –ALA (0, 0.025, 0.05, 0.1, 0.2, 0.4 mM) (A); hemin (0, 0.5, 1, 2, 3 μg/mL)(B). After the sf9 cells were harvested 72h after infection, ECOD of prepared cell lysate proteins was determined using 7-EC as substrate. In A, B the activity was expressed as relative folds compared with the blank

controls (without addition of ALA or hemin). The results are shown as the mean ± S.E (n≥3). Student's t Test was used for significance analysis. *P<0.05; **P<0.01; ***P<0.001.

Figure S5. P450 content of cell lysate with different CYP9M10 MOI infection and ECOD activity of cell lysate with co-expression of different MOI of CYP9M10 and CPR. A, Sf9 cells were infected with different CYP9M10 (MOI=0.25, 0.5, 1, 2, 3) with 1μg/mL hemin and 0.1mM 5-ALA ) in 25cm$^2$ suspension flask. With CYP9M10 MOI=0, no P450 peak was observed at around 450nm. B, co-expression CYP9M10 (1.0 pfu/cell) with different CPR (MOI=1, 0.5, 0.2, 0.1, 0.05, 0). C, ECOD activity of cell lysate with co-expression of different MOI of CYP9M10 and CPR with fixed MOI ratio of 10. Control represents no infection on sf9 cells. In all these experiments, 1μg/mL hemin and 0.1mM 5-ALA were added at the 24h post infection. Cell lysate were used for ECOD activity measurement. The results are shown as the mean ± S.E (n≥3). Statistical significance is represented by P ≤0.05 with different alphabet letters (a or b) using One-way ANOVA.

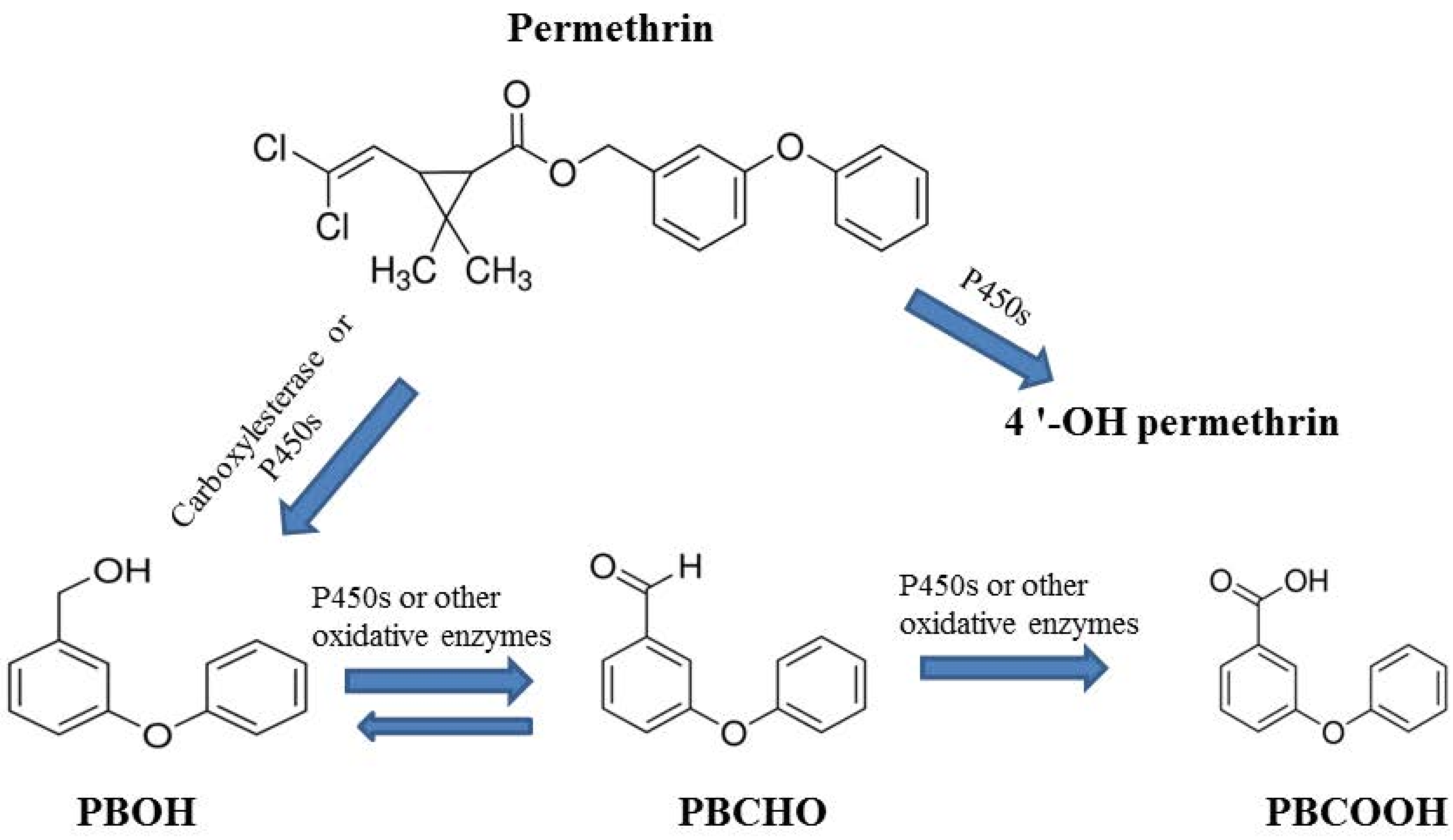
Permethrin
Cl
Cl
O
O
O
H3C CH3
P450s
4 '-OH permethrin
Carboxylesterase or
P450s
OH
PBOH
P450s or other
oxidative enzymes
O H
PBCHO
P450s or other
oxidative enzymes
O OH
PBCOOH

Figure S1

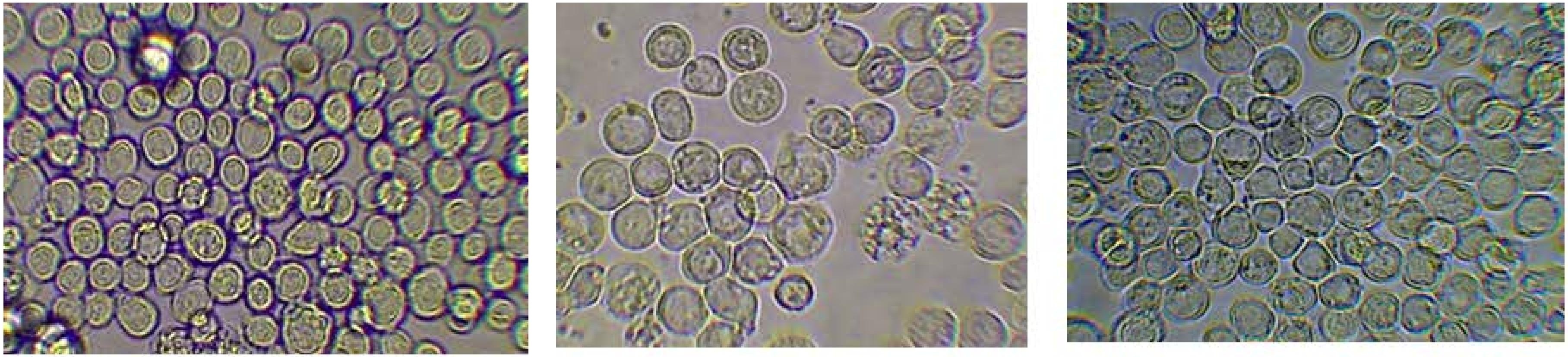

A:　empty linear virus

B: *Bv-CYP9M10* and *Bv-CPR*

C: *Bv-CYP6AA7* and *Bv-CPR*

Figure S2

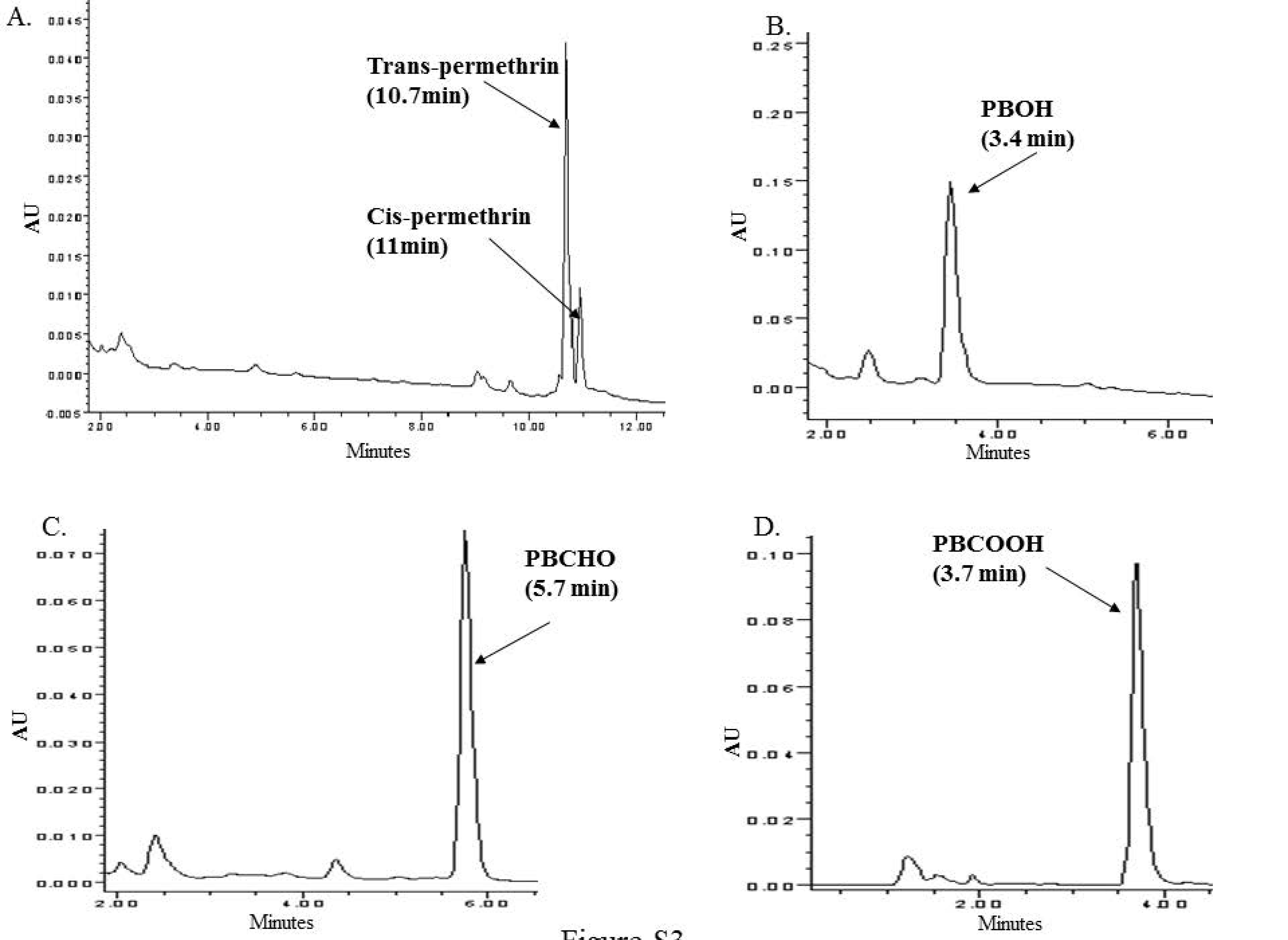

A.
Trans-permethrin
(10.7min)
Cis-permethrin
(11min)
AU
Minutes
B.
PBOH
(3.4 min)
AU
Minutes
C.
PBCHO
(5.7 min)
AU
Minutes
D.
PBCOOH
(3.7 min)
AU
Minutes


Figure S3

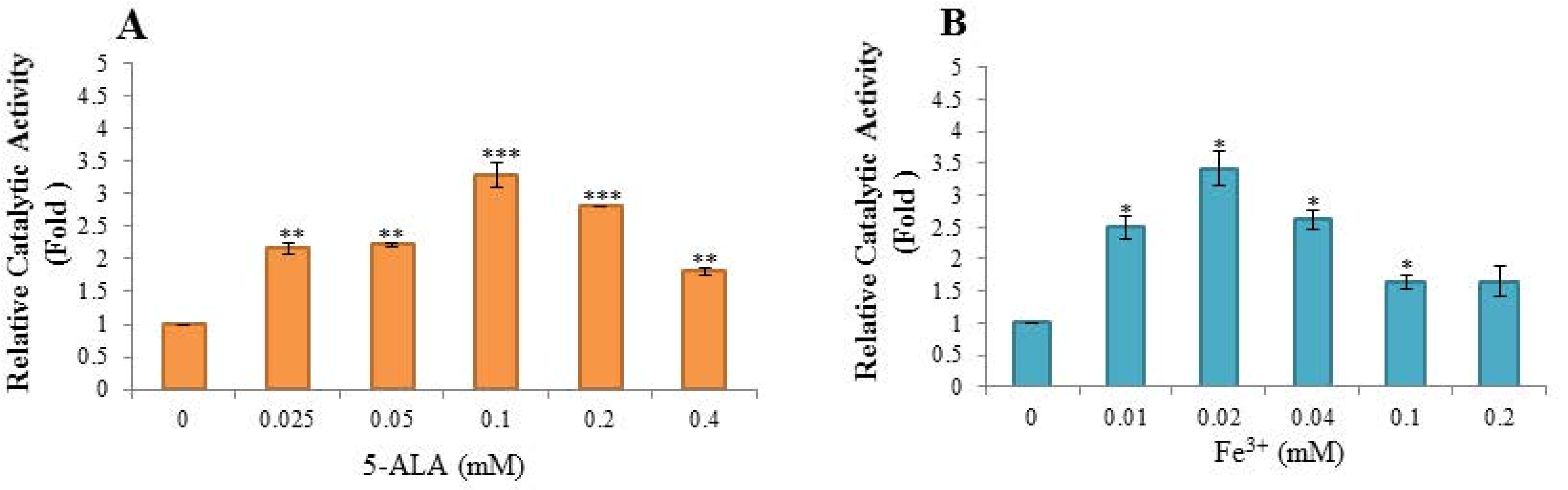
A
Relative Catalytic Activity (Fold )
5
4.5
4
3.5
3
2.5
2
1.5
1
0.5
0
**
**
***
***
**
0
0.025
0.05
0.1
0.2
0.4
5-ALA (mM)
B
Relative Catalytic Activity (Fold )
5
4.5
4
3.5
3
2.5
2
1.5
1
0.5
0
*
*
*
*
0
0.01
0.02
0.04
0.1
0.2
Fe3+ (mM)

Figure S4

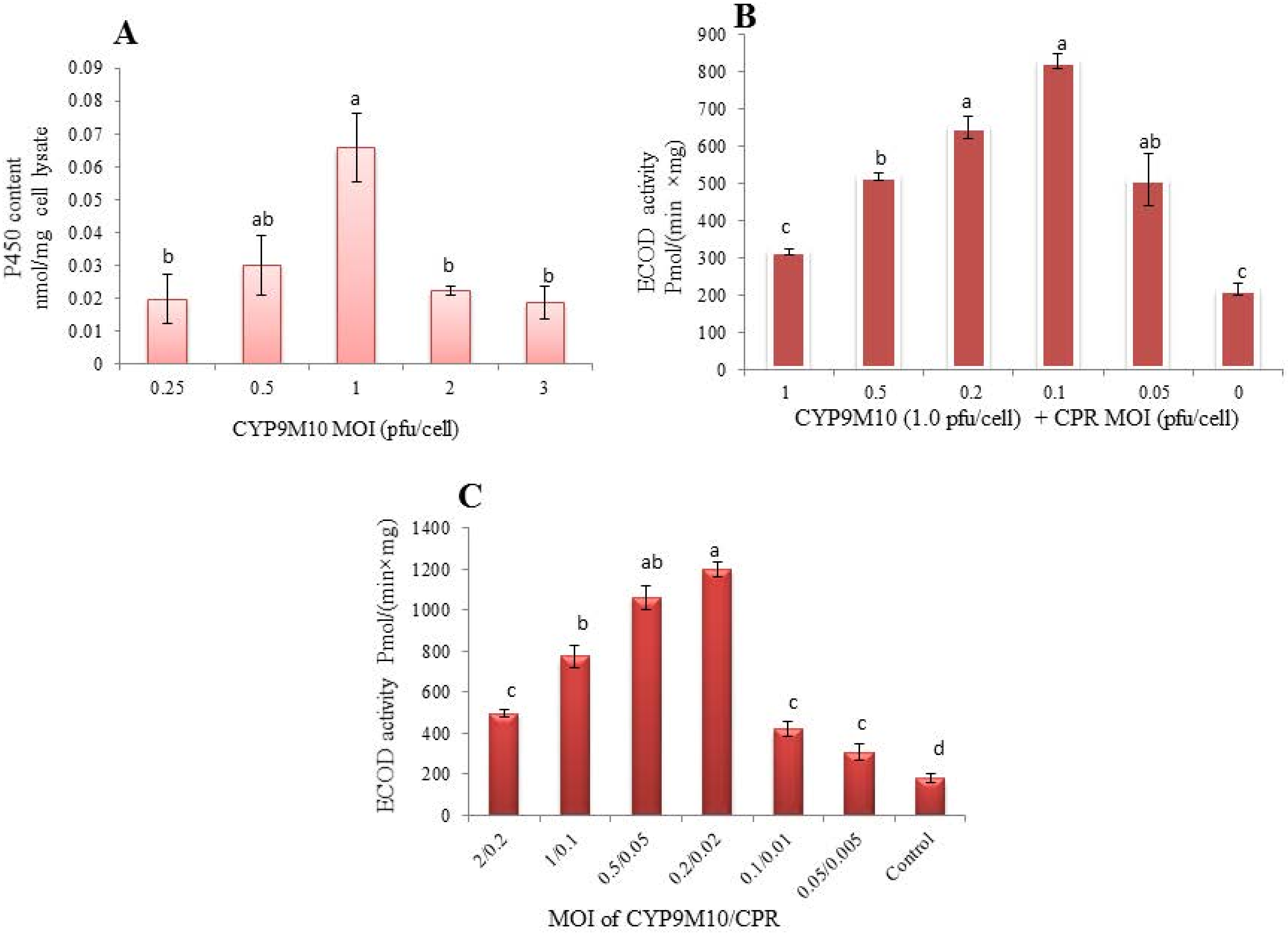

A
P450 content nmol/mg cell lysate
0.09
0.08
0.07
0.06
0.05
0.04
0.03
0.02
0.01
0
b
ab
a
b
b
0.25
0.5
1
2
3
CYP9M10 MOI (pfu/cell)
B
ECOD activity Pmol/(min ×mg)
900
800
700
600
500
400
300
200
100
0
c
b
a
a
ab
c
1
0.5
0.2
0.1
0.05
0
CYP9M10 (1.0 pfu/cell) + CPR MOI (pfu/cell)
C
ECOD activity Pmol/(min× mg)
1400
1200
1000
800
600
400
200
0
c
b
ab
a
c
c
d
2/0.2
1/0.1
0.5/0.05
0.2/0.02
0.1/0.01
0.05/0.005
Control
MOI of CYP9M10/CPR


Figure S5
